# *IRF7* and *UNC93B1* variants in an infant with recurrent herpes simplex virus infection

**DOI:** 10.1172/JCI154016

**Published:** 2023-06-01

**Authors:** Megan H. Tucker, Wei Yu, Heather Menden, Sheng Xia, Carl F. Schreck, Margaret Gibson, Daniel Louiselle, Tomi Pastinen, Nikita Raje, Venkatesh Sampath

**Affiliations:** 1 Division of Neonatology, Department of Pediatrics,; 2 Department of Pediatrics, Genomic Medicine Center, and; 3 Division of Allergy and Immunology, Department of Pediatrics, Children’s Mercy Kansas City, University of Missouri–Kansas City, Kansas City, Missouri, USA.

**Keywords:** Genetics, Infectious disease, Molecular genetics

## Abstract

Neonatal herpes simplex virus (HSV) infection is a devastating disease with substantial morbidity and mortality. The genetic basis of susceptibility to HSV in neonates remains undefined. We evaluated a male infant with neonatal skin/eye/mouth (SEM) HSV-1 disease, who had complete recovery after acyclovir but developed HSV-1 encephalitis at 1 year of age. An immune workup showed an anergic PBMC cytokine response to TLR3 stimulation but no other TLRs. Exome sequencing identified rare missense variants in IFN-regulatory factor 7 (*IRF7*) and UNC-93 homolog B1 (*UNC93B1*). PBMC single-cell RNA-Seq done during childhood revealed decreased expression of several innate immune genes and a repressed TLR3 pathway signature at baseline in several immune cell populations, including CD14 monocytes. Functional studies in fibroblasts and human leukemia monocytic THP1 cells showed that both variants individually suppressed TLR3-driven IRF3 transcriptional activity and the type I IFN response in vitro. Furthermore, fibroblasts expressing the *IRF7* and *UNC93B1* variants had higher intracellular viral titers with blunting of the type I IFN response upon HSV-1 challenge. This study reports an infant with recurrent HSV-1 disease complicated by encephalitis associated with deleterious variants in the *IRF7* and *UNC93B1* genes. Our results suggest that TLR3 pathway mutations may predispose neonates to recurrent, severe HSV.

## Graphical abstract

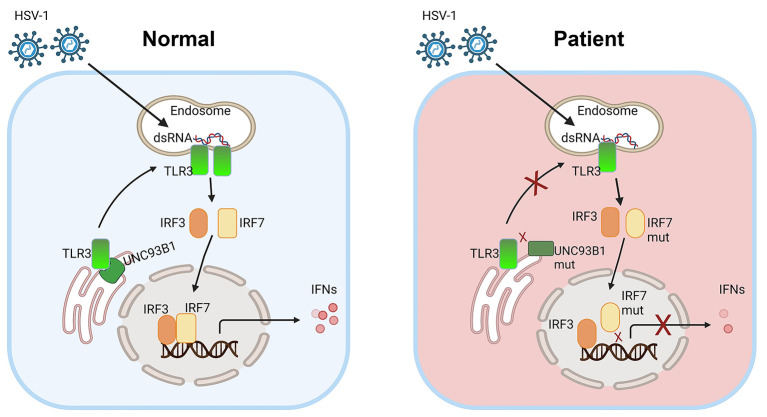

## Introduction

Neonatal herpes simplex virus (HSV) presents in neonates in the first 28 days of life as 1 of 3 subtypes: skin/eye/mouth (SEM), CNS, or disseminated disease. SEM disease is localized to the skin or mucosal membranes, and it is the most common presentation of neonatal HSV. CNS disease is infection localized to the CNS with or without SEM involvement. Disseminated disease is the most severe form and is defined as the involvement of multiple organ systems, most commonly the lungs and the liver. CNS and SEM involvement can also be present in disseminated disease (1). Despite advancements in the diagnosis and treatment of neonatal HSV, the incidence continues to increase, and the morbidity and mortality rates remain high despite treatment with acyclovir (1, 2).

HSV infection is common in adult and pediatric patients, with the estimated seroprevalence of HSV-1 at 47.8% and of HSV-2 at 11.9% in persons aged 14–49 years during 2015–2016 in the United States (3). Although seropositivity is common, primary HSV infections in adults and children are usually asymptomatic (1). However, a small minority of patients present with herpes simplex encephalitis (HSE) due to primary or recurrent HSV-1 infection. Despite the rarity of HSE, HSV remains the most common cause of sporadic encephalitis in Western countries (4). The vulnerability of selective individuals to HSV-1-induced encephalitis remained unexplained until Casrouge et al. identified a genetic defect in the TLR3-mediated IFN response in patients with HSE (5, 6).

The herpes viruses are large, enveloped, dsDNA viruses (1). The family of innate immune receptors called TLRs constitute a major component of the immune response to HSV. TLR3 receptors recognize the dsRNA produced by HSV during replication and activate a downstream signaling pathway that ultimately results in a type I IFN response (7, 8). In immunocompetent adults, HSV preferentially targets the CNS without involving other organs or causing systemic disease (4). The proclivity for HSV to selectively cause encephalitis arises from the dependency of the CNS on intact TLR3 signaling for protection against HSV, whereas this pathway is not obligately required for protection against HSV in other organ systems (9, 10). Several studies of adult and pediatric populations show that deleterious variants in the TLR3 pathway genes such as *TLR3*, TIR domain–containing, adaptor-inducing IFN-β (*TRIF*), UNC-93 homolog B1 (*UNC93B1*), and IFN-regulatory factor 3 (*IRF3*), among others, increase susceptibility to HSE (5, 6, 11, 12). However, few studies have investigated the genetic basis of neonatal susceptibility to HSV.

The newborn infant differs substantially from older children and adults, in that several arms of the adaptive and innate immune system are immature, leaving them potentially vulnerable to systemic HSV disease in addition to CNS disease (13, 14). Recently, our team identified deleterious or potentially deleterious variants in TLR3 pathway genes in neonates diagnosed with HSE or systemic HSV infections (15). Although in this pilot study we did not perform in vivo or in vitro functional analyses, it raised questions related to the genes that may contribute to neonatal HSV susceptibility, the course of the disease, and the potential for HSV recurrence in infants diagnosed with neonatal HSV. We hypothesized that deleterious variants in TLR3 pathway genes increase vulnerability to neonatal HSV disease phenotypes and that these genetic variants may predispose infants to recurrent HSV. Here, we report on an infant who recovered from neonatal SEM disease and developed HSE at 1 year of age after discontinuation of acyclovir prophylaxis. A complete immunogenetics evaluation revealed deleterious variants in the *IRF7* and *UNC93B1* genes that resulted in loss of TLR3 responsiveness in vivo and in vitro, repressed type I IFN responses, and impaired viral clearance in vitro.

## Results

### Clinical course of the proband.

The infant presented to the emergency department on day 7 of life with a worsening vesicular rash, which started on his back and spread to his chest. A full infectious workup was performed including tests for HSV. There was no maternal history of HSV infection. HSV cultures of the vesicular lesions, rectum, and mouth returned positive for HSV-1. HSV PCR of the cerebrospinal fluid (CSF) returned negative. HSV PCR of the blood was positive, with 76,000 copies/mL HSV-1. The patient did not demonstrate any other signs of disseminated HSV such as liver involvement. However, because of the diffuse nature of his vesicular rash, he received 21 days of i.v. acyclovir at 20 mg/kg/dose 3 times daily and was discharged home with a 6-month prescription for oral acyclovir for suppressive therapy at 300 mg/m^2^/dose 3 times daily. He was followed by the Infectious Disease Service as an outpatient and was prescribed weight-adjusted prophylactic acyclovir for his growth as needed. He was lost to follow up at 4 months of age. When the child was 1 year old, his mother found him in bed with tonic-clonic seizure activity. He was febrile (maximum temperature, 39.6°C) on evaluation, which was initially attributed to testing positive for multiple respiratory viruses including adenovirus, coronavirus, and rhino/enterovirus. The infant was admitted to our pediatric intensive care unit for status epilepticus and was found to have HSE with CSF positive for HSV-1. He was also noted to have 500 copies/mL HSV-1 on plasma quantitative PCR (qPCR). Brain MRI revealed findings consistent with diffuse herpes encephalitis, which was worse in the left temporal lobe, right shift of the septum pellucidum, and left uncal herniation. He was treated with i.v. acyclovir at 15 mg/kg/dose 3 times daily for 21 days, followed by oral lifelong prophylaxis at 20 mg/kg/dose 3 times daily. To control his refractory seizures, he required a midazolam drip and later a 48-hour pentobarbital-induced coma. After adequate seizure control was achieved and respiratory support was weaned, the medical team transferred the patient to the Rehabilitation Service, where he stayed for 2 months prior to his discharge home. The patient had severe loss of motor function, cognition, tone, mobility, and communication.

### An immunological workup showed attenuated PBMC responses to TLR3 stimulation.

**Figure 1:**
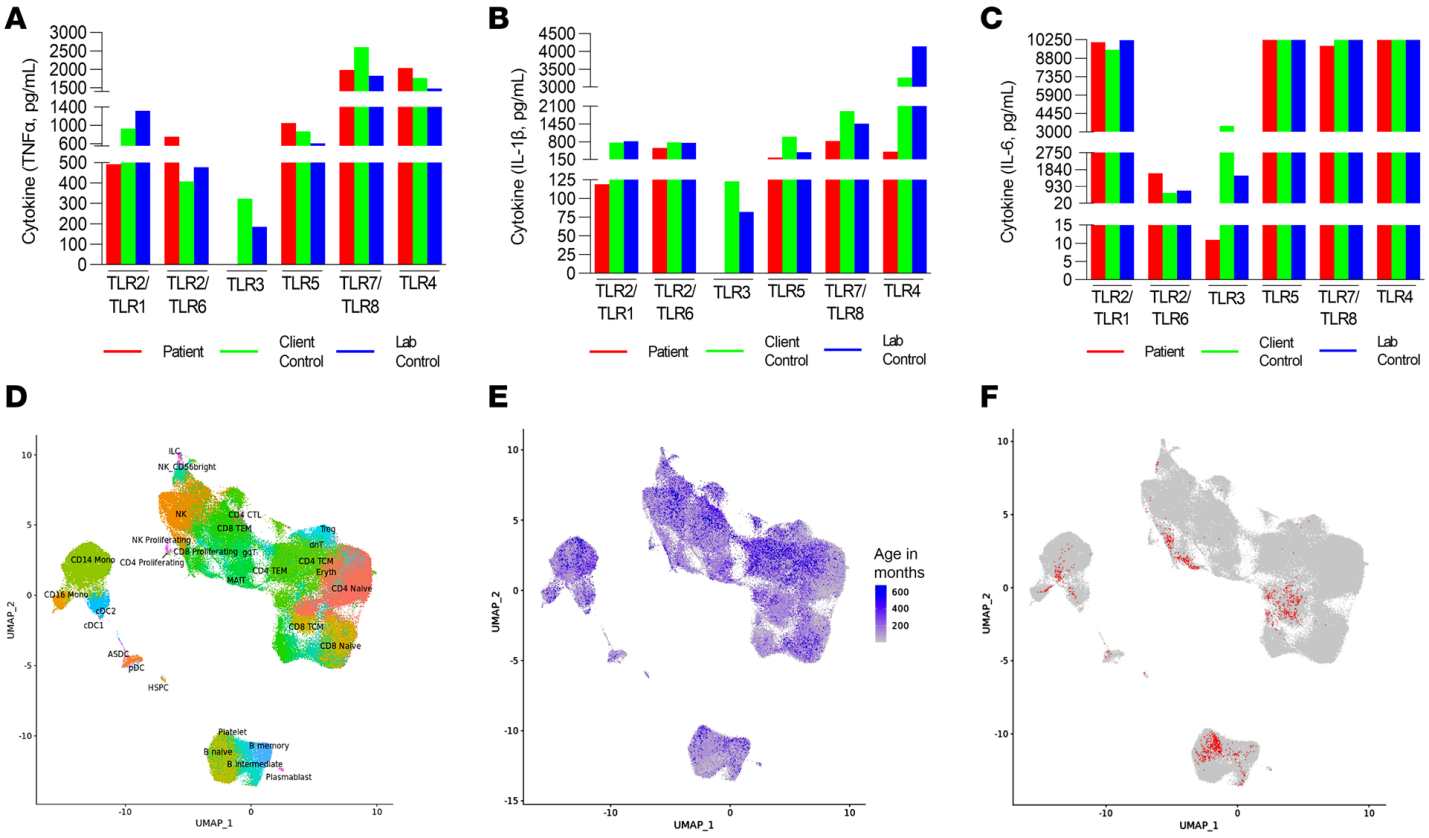
Proband’s PBMC responses to TLR ligands at 1 year of age and scRNA-Seq analysis of peripheral blood immune population at 7 years of age. (**A**–**C**) ELISA results (see Supplemental Figure 1) showing TNF-α, IL-1β, and IL-6 cytokine responses to various TLR ligands in PBMCs from the proband and controls, demonstrating anergy to TLR3 [poly(I:C)] stimulation in the proband’s PBMCs. Comparable PBMC responses to other TLR ligands were observed. TLR ligands: PAM3CSK4 (TLR1, TLR2); zymosan (TLR 2, TLR6); poly(I:C) (TLR3); flagellin (TLR5); CLO97 (TLR7, TLR8); LPS (TLR4). (**D**) UMAP of immune cell populations labeled by scRNA-Seq in 364 control samples. (**E**) UMAP showing the ages of the individuals (in months) for each of the immune cell populations identified in the control population. (**F**) UMAP of the infant’s immune cell subset projected on top of the UMAP of the control cell population.

The medical team performed an immunological workup. Flow cytometry initially showed T cell lymphopenia and NK cell lymphopenia with a normal B cell count. Naive Th and naive cytotoxic T cell counts were low. The exact laboratory values are shown in Table 1. T cell receptor excision circles were normal, at 1.09 × 10^10^ copies. Mitogen and antigen *Candida* stimulation was normal, indicating normal T cell function. The patient had normal granulocyte oxidative burst and normal NK cell function. His IgG was borderline low, which was likely due to his acute illness, and he received 1 dose of i.v. Ig. His IgM, IgA, and IgE levels were normal. Subsequent IgG levels, NK cell count, and T cell counts were normal when repeated 1 month after the acute phase of the illness. The patient exhibited an antibody response to tetanus in the intermediate range (0.04 IU/L) and in the low range to *Haemophilus influenzae* (<0.11 mg/L) related to impaired antibody formation or simply to waning immunity. The HIV antibody screen was nonreactive. The child’s PBMC functional response to specific TLR ligands was evaluated by a Clinical Laboratory Improvement Amendments–certified (CLIA-certified)laboratory (ARUP Laboratories). This evaluation showed severely attenuated IL-1β, IL-6, and TNF-α induction with TLR3 [poly(I:C)] stimulation, but normal responsiveness to TLR1/-2, TLR2/-6, TLR4, TLR5, and TLR7/-8 ligands when compared with simultaneously run controls (Figure 1, A–C, and Supplemental Table 1; supplemental material available online with this article; https://doi.org/10.1172/JCI154016DS1). Clinical exome sequencing with targeted analysis of known HSV susceptibility and immune genes revealed heterozygous missense variants in *IRF7* (p.Arg113Pro; c.338G>C) and *UNC93B1* (p.Pro404Ser; c.1210C>T).

### Single cell RNA-Seq of PBMCs revealed repressed innate immune response signatures.

To determine whether identified mutations altered the immune cell transcriptome at baseline, we performed single-cell RNA-Seq (scRNA-Seq) of PBMCs when our patient was 83 months old and did not have an acute illness. We leveraged our existing Genomic Answers for Kids (GA4K) PBMC scRNA-Seq data set from 364 individuals aged 1 month to 25 years as controls. We identified 4 major immune cell populations (B cells, T cells, monocytes, and DCs) as well as several other subpopulations (Figure 1, D and E, and Table 2) following an analysis framework, as described before (16, 17). Thirty clusters of immune cells that grouped into the 4 major cell populations, as above, were defined for the patient (Figure 1F and Table 2). The distribution of different PBMC populations with cell numbers is shown for our control cohort (*n* = 364) and our proband (Table 2). Since our control population had a very large age range (1 month to 25 years), there was more heterogeneity in this cohort. We then randomly split each of the 364 control PBMC populations into 2 halves (A and B) and did likewise with our proband’s PBMC scRNA-Seq data. Data from set B are shown in Figures 1 and Table 2, and data from set A are provided in Supplemental Figure 1. Comparison of the top 25 differentially expressed genes between the A and B subsets did not reveal major differences.

**Figure 2:**
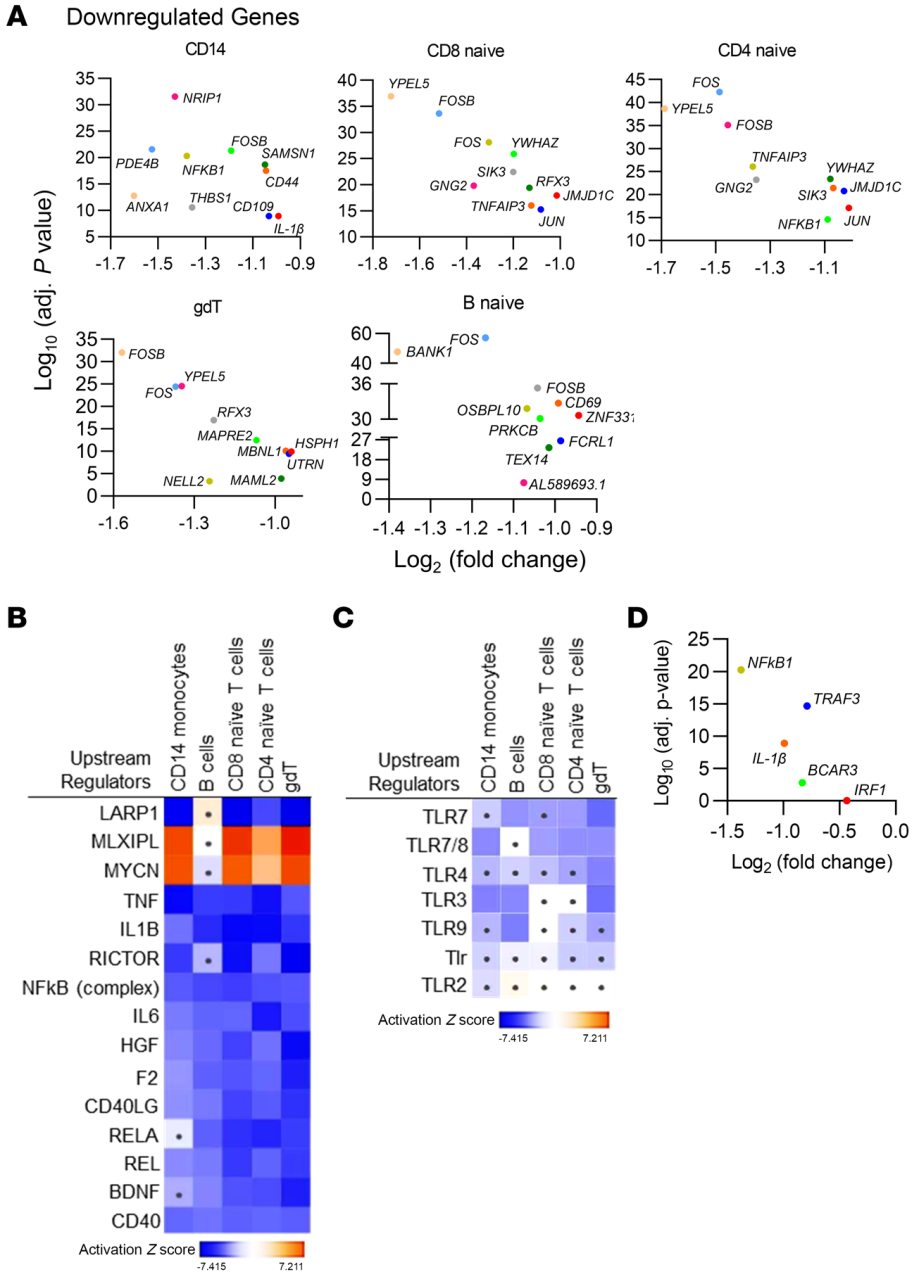
Analysis of scRNA-Seq transcriptome data for proband versus controls. (**A**) Depiction of genes repressed in CD14, CD4-naive, CD8-naive, gDT, and CD4-naive cell populations for the proband compared with controls. Data shown are only from approximately half of the cells for the control and half of the cells for the proband. The *x* axis represents log_2_-fold gene expression, and the *y* axis represents the log_10_-adjusted (adj.) *P* value. (**B**) Heatmap showing the major upstream regulators predicted by IPA to be induced or suppressed in the proband compared with controls in the major peripheral blood cell types. A dot in the center of the box represents nonsignificant results. (**C**) Heatmap illustrating IPA predictions of gene expression changes relating to specific TLR pathways in different cell types in the proband compared with the control. A dot in the center of the box represents nonsignificant results. (**D**) TLR3 pathway–related genes repressed in CD14 monocytes in proband compared with controls. Data shown are only from exactly one-half of the CD14 monocyte population for our proband and controls. The *x* axis represents log_2_-fold gene expression, and the *y* axis represents the log_10_-adjusted *P* value. For the scRNA-Seq data, a Wilcoxon rank-sum test with Bonferroni’s correction was applied for analysis.

We focused our analysis on CD14 monocytes, B naive cells, CD4-naive T cells, CD8-naive T cells, and γδ T cells, we had more than 100 cells of each type in our proband. The top 10 genes that were downregulated by 50% or more in each of these clusters are shown in Figure 2A. Most prominently, several components of the activator protein 1 (AP-1) complex such as *FOS*, *FOSB*, and *JUN* and of NF-κB, such as *NFKB1*, were downregulated. Because AP-1 and NF-κB are transcriptional regulators of cytokine responses to stress and bacterial and viral infections, the proband’s transcriptomic signature suggested a potential for a repressed immune response (18, 19). Consistent with this, Ingenuity Pathway Analysis (IPA) (Figure 2B) suggested a repression of upstream regulators of NF-κB, IL-6, IL-1β, and TNF-α signaling and of other immune pathways across the 4 cell types in our proband. As the child’s PBMC responses to TLR ligands suggested selective inhibition of responses to TLR3 ligands, we postulated that, even in the unstimulated state, we would be able to detect signatures of TLR3 pathway suppression. Specific analysis of TLR pathways indicated an inhibition of TLR3 and other endosomal virus–sensing TLR pathways in the cell populations of interest but not of bacteria-sensing TLR4 or TLR2 pathways (Figure 2C and Supplemental Figure 1, B–D). Genes that were downregulated by more than 50% in CD14 monocytes, which indicated potential repression of TLR3 signaling, included *NFKB1*, *IL1B*, and TNF receptor–associated factor 3 (*TRAF3*) (Figure 2D and Supplemental Figure 1C). TRAF3 is a key adaptor that is important for antiviral immunity, and mutations in *TRAF3* have been shown to be associated with HSE in adults (20, 21). In summary, our scRNA-Seq data indicate impaired innate immune responses specifically to endosomal virus–sensing pathways, and this was consistent with the patient’s anergic PBMC responses to the TLR3 ligand poly(I:C).

### Functional annotation and analysis of the IRF7 missense variant.

**Figure 3:**
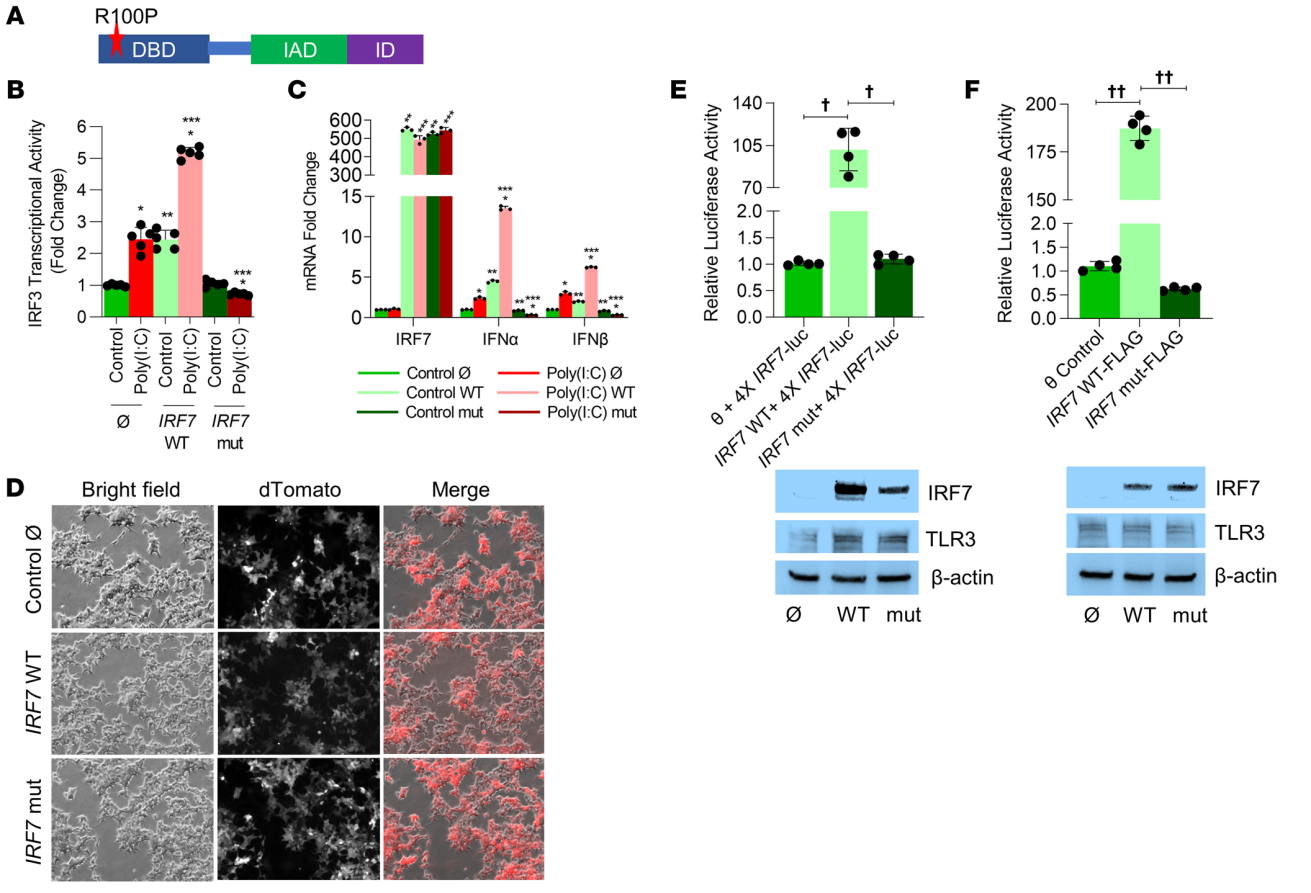
The *IRF7* variant (p.R100P) abolishes poly(I:C)-induced IRF3 transcriptional activity, type I IFN expression, and *IRF7* DNA binding in THP1 and HEK293T cells. (**A**) Model of *IRF7* mapping of the p.R100P variant on the DNA-binding domain (DBD). IAD, IRF association domain; ID, inhibitory domain. (**B** and **C**) Ø and *IRF7* reference WT and mut proteins [mut plasmids were transfected into THP1 cells prior to treatment with 1 μg/mL poly(I:C) for 24 hours]. (**B**) IRF3 transcriptional activity was measured by quantifying luciferase in culture supernatants obtained from THP1 cells transfected with plasmids and treated with poly(I:C). *Control versus poly(I:C); **poly(I:C) Ø versus poly(I:C) plasmid; ***control Ø versus control plasmid; *P* < 0.05 (all). *n* = 5. (**C**) RNA expression of *IRF7*, *IFNA*, and *IFNB* was quantified in THP1 cell lysates obtained after the above treatments by qPCR. *Control versus poly(I:C); **poly(I:C) Ø versus poly(I:C) plasmid; ***control Ø versus control plasmid; *P* < 0.05 (all). *n* = 3. (**D**) Bright-field and fluorescence images of HEK293T cell lines stably expressing control Ø, IRF7 WT, and IRF7 mut, respectively. Original magnification, ×100. (**E**) HEK293T were transfected with pGL4-4X IRF7-minP-Luc, pRL-TK (thymidine kinase promoter–*Renilla* luciferase reporter plasmid), CFP-TLR3, Ø, and *IRF7* WT or mut plasmids for 2 days. Cells were lysed and a luciferase assay was performed. Part of the cell lysate was used to quantify expression of IRF7 WT and mut protein by immunoblotting. ^†^*P* < 0.0001. *n* = 4. (**F**) HEK293T cell lines stably expressing Ø, WT, or mut FLAG-tagged IRF7 protein were transfected with pGL4-4X IRF7-minP-Luc, pRL-TK, and CFP-TLR3 for 2 days. Cell lysates were used for luciferase assay and protein expression studies. ^††^*P* < 0.001. *n* = 4. A Mann-Whitney *U* test or ANOVA with post hoc Tukey’s test was used for analysis after testing for normality.

Our proband was heterozygous for a rare missense variant in the *IRF7* (p.Arg113Pro; c.338G>C) gene, with a minor allele frequency (MAF) of 0.00007 (National Library of Medicine dbSNP, https://www.ncbi.nlm.nih.gov/snp/rs752300122). The variant was predicted to be deleterious using SIFT, Polyphen II, and MAPP. IRF7 is a transcription factor, and its activation is required for an effective type I IFN response to viruses (22). To determine whether the *IRF7* variant alters TLR3 responsiveness, we subcloned WT and *IRF7* variant alleles into expression vectors and examined functionality in a commercially available monocyte-macrophage THP1 cell line, in which luciferase activity is driven by IRF3 transcriptional activity (InvivoGen). We used poly(I:C), a synthetic analog of dsRNA and activator of TLR3 signaling, to stimulate THP1 cells transfected with WT *IRF7* or *IRF7* (p.Arg113Pro; c.338G>C) alleles (Figure 3B). We found that WT *IRF7* enhanced the baseline responsiveness to poly(I:C), implying that *IRF7* amplifies TLR3-induced IRF3 transcriptional activity, as has been shown by studies noting that IRF7-IRF3 heterodimers regulate type I IFN responsiveness (22). The *IRF7* (p.Arg113Pro; c.338G>C) variant suppressed even baseline poly(I:C)-induced IRF3 transcriptional activity. We next examined TLR3-stimulated type I IFN responsiveness with the WT and variant *IRF7* alleles. Poly(I:C)-stimulated *IFNA* and *IFNB* gene expression induced by WT *IRF7* was abolished in cells transfected with the *IRF7* (p.Arg113Pro; c.338G>C) variant (Figure 3C). Our data are consistent with those reported by Honda et al., who demonstrated using mice and fibroblasts that *IRF7* is obligately required for a TLR-mediated myeloid differentiation factor 88–independent antiviral type I IFN response (23).

The *IRF7* variant localizes to the nuclear-binding domain of IRF7 (Figure 3A) and is important for its transcriptional activity. We hypothesized that the *IRF7* variant would impair IRF7 binding to the consensus binding elements present in the promoter region of downstream target genes. To test this, we constructed a reporter containing a 4× human *IRF7* consensus site attached to firefly luciferase. We created stable HEK293T cell lines expressing the WT or *IRF7* variant alleles (Figure 3D). We transfected the consensus *IRF7* reporter into the stable cell lines and discovered that there was strong induction of luciferase with the *IRF7* WT allele but no activity with the *IRF7* (p.Arg113Pro; c.338G>C) variant (Figure 3F). These results were also confirmed with transient transfection experiments in HEK293 cells (Figure 3E). These data reveal that the *IRF7* variant identified in our proband represses the transcriptional activity of IRF7 and abolishes poly(I:C)-induced IRF3 transcriptional activity and type I IFN responses.

### Functional annotation and analysis of the UNC93B1 missense variant.

**Figure 4:**
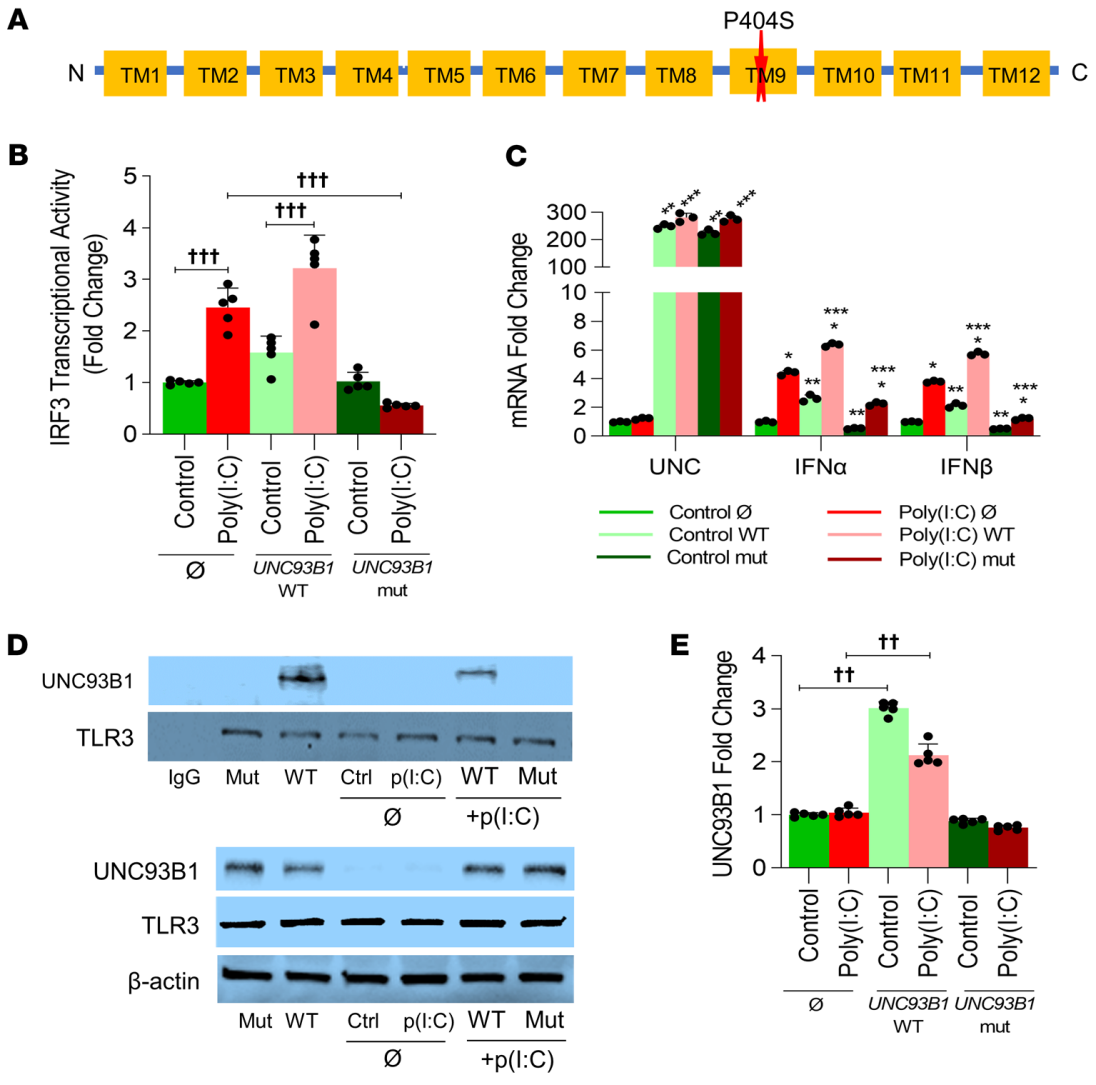
Functional analysis of the *UNC93B1* variant (p.P404S) in THP1 cells showing that the *UNC93B1* variant abolishes poly(I:C)-induced IRF3 transcriptional activity, type I IFN expression, and binding to the TLR3 protein. (**A**) Model of *UNC93B1* showing localization of the p.P404S variant to transmembrane domain 9 (TM9), implicated in physical binding to TLR3. (**B**–**E**) THP1 cells were transfected with empty plasmid (Ø), *UNC93B1* WT, and mut plasmids prior to treatment with 1 μg/mL poly(I:C) for 24 hours. (**B**) IRF3 transcriptional activity was measured by quantifying luciferase in culture supernatants obtained from THP1 cells transfected with the indicated plasmids following 24 hours of poly(I:C) treatment. ^†††^*P* < 0.05. *n* = 5. (**C**) mRNA expression of *UNC93B1* (UNC), *IFNA*, and *IFNB* was quantified by qPCR in THP1 cell lysates obtained after the above treatments. *Control versus poly(I:C); **poly(I:C) Ø versus poly(I:C) plasmid; ***control Ø versus control plasmid; *P* < 0.05 (all). *n* = 3. (**D** and **E**) TLR3 was immunoprecipitated from THP1 clarified lysates (**D**) to demonstrate that binding of UNC93B1 to TLR3 was repressed with the *UNC93B1* variant (top panel). Bottom panel shows expression of transfected WT and mut *UNC93B1* alleles in THP1 cells. (**E**) Densitometric quantification of the Western blots shown in **D.** ^††^*P* < 0.001. *n* = 5. ANOVA with Tukey’s test was used for statistical analysis.

Our patient was also heterozygous for a missense mutation in the *UNC93B1* (p.Pro404Ser; c.1210C>T) gene, which has a mean allele frequency of 0.0006 (NLM dbSNP, https://www.ncbi.nlm.nih.gov/snp/rs377021545) (Figure 4A). UNC93B1 is essential for the trafficking of nucleic acid–sensing TLRs (including TLR3) to endosomes as well as for the activation and stabilization of TLR3 (24). Luciferase assays of THP1 cells showed that, while WT *UNC93B1* induced TLR3-driven IRF3 transcriptional activity in response to poly(I:C), the *UNC93B1* (p.Pro404Ser; c.1210C>T) allele repressed IRF3 transcriptional activity (Figure 4B). Similarly, *IFNA* and *IFNB* expression induced by poly(I:C) was enhanced by the WT *UNC93B1* allele, but strongly suppressed by the *UNC93B1* variant allele (Figure 4C). As the *UNC93B1* variant localizes to the TLR-binding domain of *UNC93B1*, we postulated that binding to TLR3, which is required for the trafficking of TLR3 from the endoplasmic reticulum to endosomes, would be disrupted. Co-IP studies performed in THP1 cells revealed that, while the WT *UNC93B1* bound to TLR3, the *UNC93B1* variant did not bind to TLR3 (Figure 4, D and E). These data suggest that the *UNC93B1* variant decreases binding to TLR3, resulting in loss of poly(I:C)-mediated IRF3 transcriptional activity and type I IFN secretion.

### Functional annotation and analysis of the combined IRF7 and UNC93B1 missense variants.

**Figure 5:**
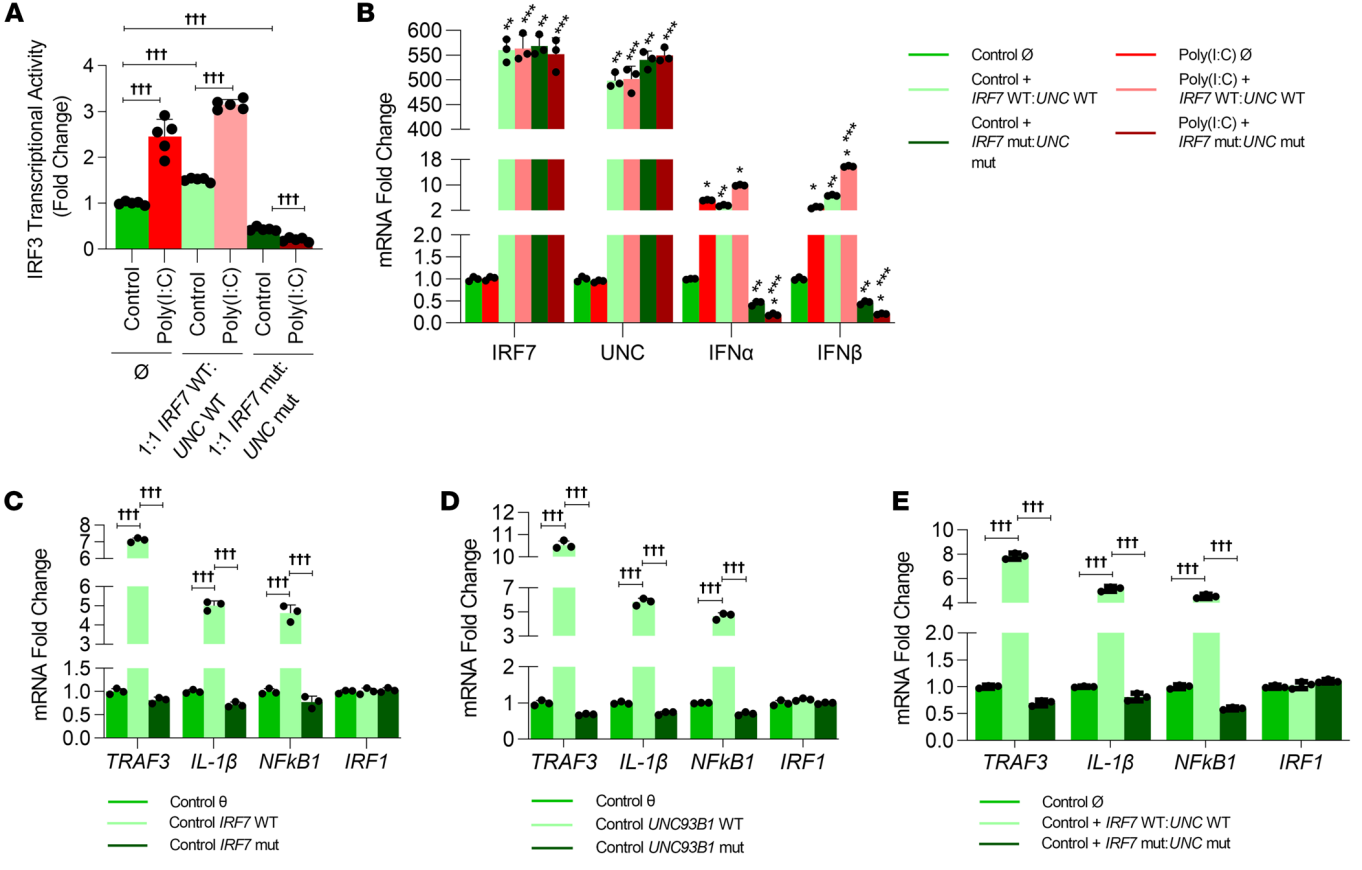
Functional analysis of the *UNC93B1* variant p.P404S and the *IRF7* variant p.R100P in THP1 cells shows anergic responses to stimulation with poly(I:C). (**A**) IRF3 transcriptional activity was measured by quantifying luciferase in culture supernatants obtained from THP1 cells transfected with the indicated plasmids and treated with poly(I:C). *P < 0.05. *n* = 5. (**B**) mRNA expression of *IRF7*, *UNC93B1*, *IFNA*, and *IFNB* was quantified by qPCR in THP1 cell lysates obtained after the above treatments. *Control versus poly(I:C); **poly(I:C) Ø versus poly(I:C) plasmid; ***control Ø versus control plasmid; *P* < 0.05 (all). *n* = 3. (**C**–**E**) Controls [poly(I:C)-untreated] from the THP1 cells transfected with IRF7 (**C**), UNC93B1 (**D**), both reference alleles and both variant plasmids (**E**) were used for quantification of TRAF3, IL-1β, NF-κB1, and IRF1. *n* = 3. †††*P* < 0.05. A Mann-Whitney *U* test or ANOVA with post hoc Tukey’s test was used for statistical analysis after testing for normality of distribution.

We next pursued studies to examine the deleterious effect of both variants combined in our cell culture system to mimic the clinical scenario in our proband. We transfected both the WT and variant alleles of *IRF7* and *UNC93B1* into THP1 cells, followed by challenge with poly(I:C). Poly(I:C)-mediated *IRF3* transcriptional activity (Figure 5A) and type I IFN cytokine expression (Figure 5B) were severely suppressed, recapitulating the anergic PBMC responses to poly(I:C) noted in our patient. Our scRNA-Seq analysis of PBMCs identified decreased expression of *IL1B*, *NFKB1*, and *TRAF3* in CD14 monocytes. We therefore probed to determine whether the baseline expression of these genes was altered with the *UNC93B1* and *IRF7* variants. Expression of the WT *UNC93B1* and *IRF7* alleles induced baseline expression of *IL1B*, *NFKB1*, and *TRAF3* independently or in combination in THP1 monocytes (Figure 5, C–E). Interestingly, the variant *UNC93B1* and *IRF7* alleles did not induce the above genes, but rather resulted in a modest reduction in expression (Figure 5, C–E). Expression of *IRF1*, which was not altered in the scRNA-Seq analysis of the CD14 monocyte population in our proband, remained unchanged with expression of the reference or variant *UNC93B1* and *IRF7* alleles in THP1 cells (Figure 5, C–E). These data demonstrate that the *UNC93B1* and *IRF7* variants, when combined, result in complete anergy to the TLR3 ligand poly(I:C) in THP1 cells, mimicking the anergy to poly(I:C) noted in the child’s PBMC responses. Our studies also revealed that *UNC93B1* and *IRF7* variants repressed RNA expression of *NFKB1*, *IL1B*, and *TRAF3* both in vivo and in vitro.

### IRF7 and UNC93B1 variants impair intracellular clearance/replication of HSV-1 and the type I IFN response in human fibroblasts.

**Figure 6:**
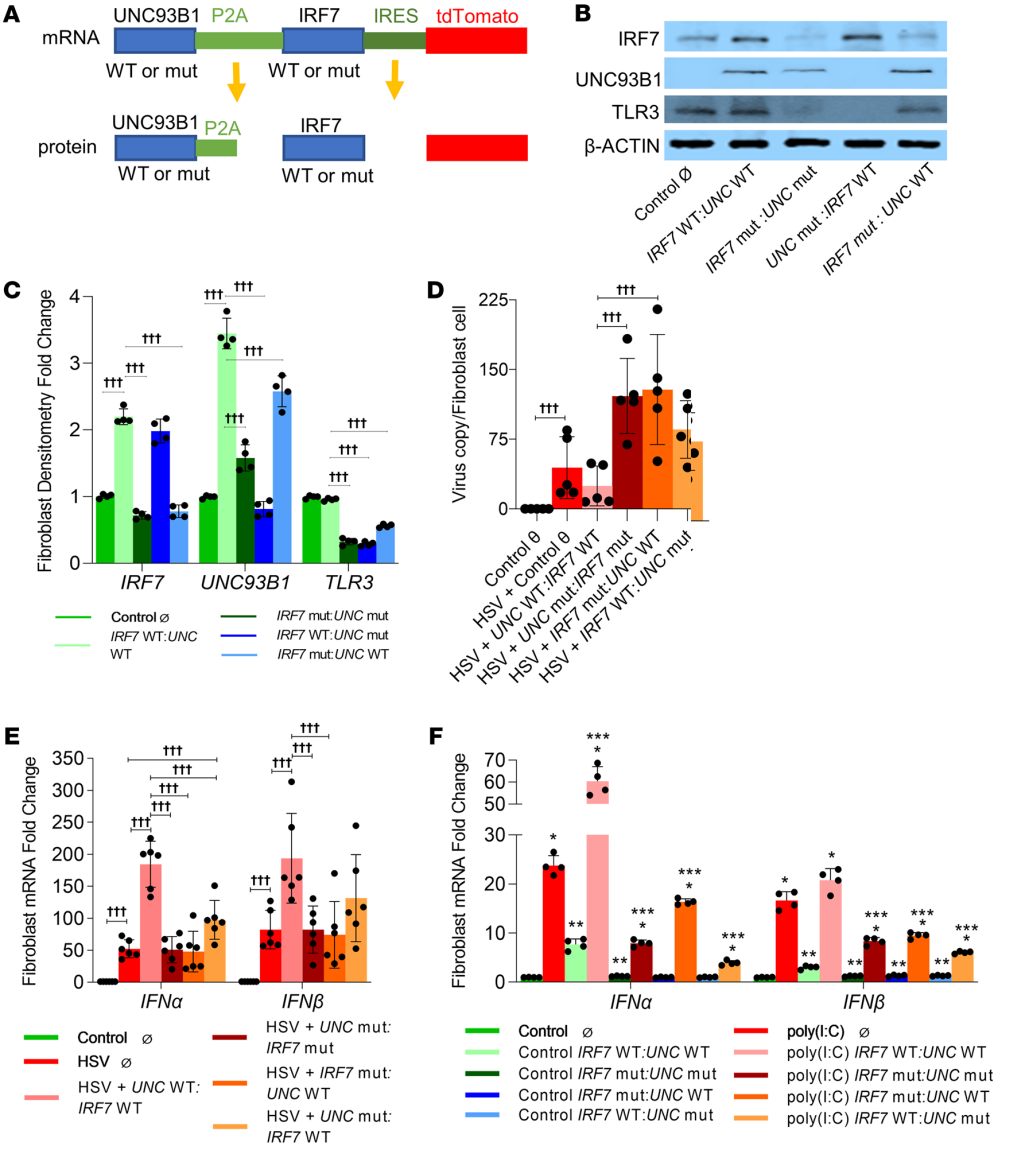
Neonatal human fibroblasts carrying the *UNC93B1* variant p.P404S and the *IRF7* variant p.R100P showed abolished responses to stimulation with poly(I:C) or HSV-1. Neonatal human fibroblasts stably expressing dTomato alone (control Ø), *UNC93B1* and *IRF7* double WT, UNC93B1 and IRF7 double-mut, the *IRF7* mut:*UNC93B1* WT, and the *UNC93B1* mut:*IRF7* WT were used in this experiment. (**A**) Graphic representation of the plasmid structure and related mature protein. (**B**) Western blot shows the expression of IRF7, UNC93B1, and TLR3 in all stable cell lines and (**C**) densitometric analysis. *n* = 4. †††*P* < 0.05. (**D**) The fibroblast cell lines were incubated with HSV-1 at a MOI of 10 for 1 hour, HSV Virus copy was quantified from the cell lines after a 16-hour infection. *n* = 5. †††*P* < 0.05. (**E**) The fibroblast cell lines were incubated with HSV-1 at MOI of 10 for 1 hour, and the resulting gene expression 6 hours after infection is shown. *n* = 6. †††*P* < 0.05. (**F**) These cell lines were incubated with 1 μg/mL poly(I:C) for 24 hours, and mRNA expression is shown for *IFNA* and *IFNB*. *Control versus poly(I:C); **poly(I:C) Ø versus poly(I:C) plasmid; ***control Ø versus control plasmid; *P* < 0.05 (all). *n* = 3. A Mann-Whitney *U* test or ANOVA with post hoc Tukey’s test was used for statistical analysis after testing for normality of distribution.

As both the *IRF7* and *UNC93B1* variants disrupted the TLR3-dependent response to extracellular stimulation with poly(I:C), we postulated that identified variants would impair the immune response to HSV-1. Human fibroblasts display a TLR3-dependent response to HSV and poly(I:C) and have previously been used to characterize functional defects in innate immune genes (5, 9). We used commercially available neonatal human dermal fibroblasts to generate human dermal fibroblast lines stably expressing both reference alleles, both variant alleles of *IRF7* and *UNC93B1*, and individual variant alleles with their counterpart reference alleles for functional analysis (Figure 6, A and B). Interestingly, we noted that TLR3 protein expression decreased significantly in fibroblast lines expressing the *UNC93B1* variant (Figure 6, B and C). This is consistent with UNC93B1’s role in binding, chaperoning, and preventing the destruction of TLR3 as reported previously (24). Our data (Figure 4E) demonstrating decreased binding of the *UNC93B1* variant to the TLR3 provide further functional corroboration. We also noted decreased expression of mutant (mut) proteins (Figure 6, B and C).

We then examined whether the identified variants would impair the host’s ability to control HSV-1 replication (25). Using qPCR to detect specific DNA sequences of HSV-1, we compared the genomic copy number within fibroblast lines expressing different combinations of variant/reference alleles 16 hours after a titrated challenge with HSV-1 particles (MOI 10) (26). Intracellular viral DNA titers decreased in cells overexpressing the reference allele by more than 40% (Figure 6D), whereas cells expressing both variant alleles had an almost 3-fold increase in viral DNA titers compared with titers in control fibroblasts (Figure 6D). Interestingly, intracellular viral DNA titers in fibroblasts expressing the *IRF7* variant with the reference *UNC93B1* allele also had elevated HSV viral DNA titers that were not quantifiably different from those of fibroblasts expressing both variants (Figure 6D). Fibroblasts expressing the *UNC93B1* variant with the reference *IRF7* allele had increased viral DNA titers compared with controls but lower titers than fibroblasts expressing the *IRF7* variant. We also examined all fibroblast lines for expression of IFNs 6 hours after HSV-1 infection. Fibroblasts with both reference alleles showed increased IFN expression compared with control fibroblasts (Figure 6E). Importantly, type I IFNs were not induced in fibroblasts expressing both the variant alleles. The IFN response was also blunted in cells expressing the *IRF7* variant plus WT *UNC93B1*, but there was induction of type I IFNs in cells expressing the *IRF7* reference allele and the *UNC93B1* variant allele (Figure 6E). These observations indicate that, while both the *IRF7* and *UNC93B1* variants are functionally deleterious, the *IRF7* variant acts in a dominant negative manner, as it is not rescued by overexpressing the *UNC93B1* reference allele. Experiments using poly(I:C) to stimulate generated fibroblast lines showed similar results, with the *UNC93B1* and *IRF7* variants resulting in partial and total loss of type I IFN responsiveness, respectively (Figure 6F).

## Discussion

Monogenic inborn errors of innate immune signaling often manifest during infancy and childhood, with susceptibility to a narrow spectrum of bacterial pathogens. Genetic defects in bacterial sensing arising from mutations in canonical TLR signaling pathway genes such as *IRAK4* and *MYD88* are established causes of primary immunodeficiency. Whether neonatal HSV infections presenting as SEM, CNS, or disseminated disease are phenotypes for inborn errors in virus-sensing TLR3 pathway genes remains unclear, although a recent genetic survey of neonatal HSV disease suggested a link (15). In this study, we identified 2 genetic variants in *IRF7* and *UNC93B1* in our proband, who had full recovery from neonatal SEM disease but had a recurrence of HSV encephalitis during infancy after stopping preventative acyclovir therapy. Using cellular, molecular, and scRNA-Seq approaches, we demonstrate that the identified variants resulted in anergic type I IFN responses to TLR3 ligands and impaired intracellular HSV-1 clearance. Our results indicate that the identified *IRF7* and *UNC93B1* variants contributed to anergic TLR3 responses and recurrent HSV in our patient.

Our patient underwent a complete immunological workup, which was largely unremarkable aside from a severe attenuated response to TLR3 stimulation. Changes in the IgG subsets and T cell population in the acute phase of HSE in the patient recovered after resolution of the acute illness as shown in Table 1. The anergic response of the child’s PBMCs to poly(I:C) was the first indication that a defect in the TLR3 signaling pathway may be responsible for our patient’s recurrent HSV infections. The parents did not consent to genetic testing, so we were unable to determine whether these rare variants arose de novo or were inherited. To investigate the effect of the identified *IRF7* and *UNC93B1* variants on the peripheral blood immune cell transcriptome, we performed scRNA-Seq of the proband’s PBMCs when he was 7 years old, was on acyclovir prophylaxis, and did not have active HSV. Although this screen represents a native expression profile, we posited that we would see defects in innate immune and TLR3 pathways. Compared with controls, our proband’s transcriptome showed significant reductions in *IL1B*, *NFKB1*, and *AP1* complex genes in several populations including CD14 monocytes. Correspondingly, IPA analysis predicted that upstream regulators of NF-κB, TNF, IL-1β, and IL-6 signaling were also repressed. Interestingly, IPA analysis showed that TLR3 and other endosomal virus–sensing TLR pathways such as TLR7 were inhibited, but not bacteria-sensing TLR2, -4, or 5 pathways (27, 28). These predictions were based on decreased expression of *TRAF3* (an adapter for viral TLR sensing), *NFKB1*, and *IL1B* (20, 21). Analysis of the PBMC transcriptome during the active infection phase would have complemented our data but was not feasible. A limitation of our analysis is that the age group of our control population was very broad but did include children ranging from 1 month old to 12 years old. Analysis of PBMC scRNA-Seq data for children 5–9 years old, 2 years older or younger than our patient, also revealed similar trends (data not shown). Increasingly, combining scRNA profiling with genetic sequencing is being used to discern cell-type–specific molecular defects in immunodeficiency (29).

IRF7 is a transcription factor that serves as a crucial regulator of the type I IFN immune response to viral infections, including HSV (22, 30). In mouse models, experiments show that *IRF7 ^–/–^* mice are unable to generate a type I IFN response, and in adult humans, reports of single-gene variants in *IRF7* have been associated with severe presentation of common viral infections (31–34). However, to the best of our knowledge, the association between an *IRF7* mutation and HSV susceptibility in humans has not been described. Ciancanelli et al. reported 2 compound heterozygous single-gene variants in *IRF7* associated with severe influenza virus infection (33). In addition, Zhang et al. recently identified inborn errors in *IRF7* in patients with life-threatening COVID 19 when compared with patients with asymptomatic or benign COVID-19 infection (32). The *IRF7* variant identified in our patient localized to its DNA-binding domain and was unable to activate the IRF7 transcriptional response, as it is not able to bind the promoters of downstream targets. This impaired IRF3 transcriptional activity and abolished the type I IFN response following TLR3 stimulation in both THP1 cells and fibroblasts (Figure 3, B and C). Our results suggest that IRF7 activation is crucial for IRF3-dependent type I IFN response and are consistent with studies showing that dimerization of IRF3/IRF7 regulates IFN-α and IFN-β expression (22). Furthermore, we also observed increased intracellular copies of HSV-1 DNA in fibroblasts expressing the *IRF7* variant both in combination with the *UNC93B1* variant or alone when compared with controls (Figure 6D). Interestingly, the inability of the *UNC93B1* reference allele to rescue the deleterious effect of the *IRF7* variant on HSV-1 replication or the type I IFN response suggests that the *IRF7* variant exerted a dominant effect. This was also confirmed by our observations that cell lines expressing both the *IRF7* and *UNC93B1* variants did not show a greater defect than did cells expressing only the *IRF7* variant. These results, in conjunction with the growing body of literature showing that monogenic defects in *IRF7* can predispose individuals to severe viral illnesses, support the hypothesis that the *IRF7* variant identified in our patient is deleterious and contributed to his recurrent HSV-1 infection.

Mutations in *UNC93B1* have been previously described in pediatric and adult patients with HSE, but are less well characterized in the neonatal population (5, 6). UNC93B1 is essential for the trafficking of nucleic acid–sensing TLRs (including TLR3) to endosomes as well as for the activation and stabilization of TLR3 (24). Our results show the *UNC93B1* variant identified in our patient disrupted the binding of UNC93B1 to TLR3 (Figure 4, D and E) and subsequently resulted in the repression of IRF3 transcriptional activity and a diminished type 1 IFN response (Figure 4, B and C). We also noted that, in our transformed fibroblast cell line, TLR3 protein expression was diminished in cell lines with the *UNC93B1* variant. In addition, intracellular viral titers were increased in fibroblasts expressing the *UNC93B1* variant with the *IRF7* reference allele or both the *UNC93B1* and *IRF7* variants (Figure 6D). Although *UNC93B1*-related HSE is typically reported as an autosomal recessive disease, autosomal dominant HSE resulting from mutations in *TRAF3* and *TLR3* has been reported (10). Our studies were not able to discern which deleterious variant contributed more to HSV susceptibility, but we suspect that TLR3 anergy arising from the combination of these variants contributed to our patient’s recurrent HSV disease. Interestingly, fibroblasts exposed to HSV-1 virus with the *UNC93B1* variant and reference *IRF7* allele had increased HSV-1 viral titers, but less so than did fibroblasts expressing the *IRF7* variant with the reference *UNC93B1* allele. Strikingly, the decreased expression of *TRAF3*, *NFKB1*, and *IL1B* noted in our proband’s scRNA-Seq data was replicated to a modest extent in the native state in our in vitro assays with both variants. This implies the conservation of deleterious effects of the variants on the type I IFN response across cell lines and suggests a broader defect in innate immune signaling that, to our knowledge, has not been recognized.

Our results clearly evince that the *IRF7* and *UNC93B1* variants identified in our child with recurrent HSV disease impaired the TLR3-mediated IRF3-dependent type I IFN response and viral clearance independently and likely contributed to this child’s clinical course. This study raises questions regarding genetic screening of neonatal HSV infections and optimal strategies for acyclovir prophylaxis. Despite the progress made in recent years in the diagnosis and treatment of HSV, the incidence of HSV diagnosis in the neonatal population continues to increase (2). Although our proband had only SEM disease as a newborn and received acyclovir prophylaxis for 6 months according to the American Academy of Pediatrics Redbook guidelines, he developed a devastating recurrence after stopping prophylaxis (1). As susceptibility to HSE persists through adulthood, and our recent report noted that approximately 50% of neonatal systemic or HSE disease might be associated with rare, deleterious missense variants in TLR3 signaling genes, a case could be made for immunogenetic screening of all infants with severe neonatal HSV (15). Furthermore, instituting a longer duration of acyclovir therapy and immune surveillance programs akin to those for infants and children with inborn errors of innate immune signaling arising from *MyD88* and *IRAK4* mutations could emerge as options for infants with neonatal HSV with identified deleterious variants (35). Although HSV-1 was detected in both the newborn’s surface cultures and later in the CSF during HSE infection, we were not able to confirm whether his HSV recurrence was related to reactivation of the primary infection or secondary to a new HSV-1 infection, as the neonatal specimens were not retained. In summary, we report a case of recurrent HSV disease in an infant with deleterious variants in *IRF7* and *UNC93B1*, which raises questions regarding genetic screening, immune workup, and long-term management of infants with neonatal HSV disease.

## Methods

We combined exome sequencing with in vivo and in vitro immune functional analysis to discover the immunogenetic basis of HSV vulnerability in a proband.

### TLR assay.

TLR function testing was performed by functional assay on PBMCs (ARUP Laboratories). TLRs were tested independently by stimulation with TLR-specific ligands. PBMC cytokine production of IL-1β, IL-6, and TNF-α was determined by multianalyte fluorescence detection. The TLR-specific ligands included PAM3CSK4, a synthetic bacterial lipoprotein (TLR2-TLR1 ligand); zymosan cell wall particles from *Saccharomyces cerevisiae* (TLR6-TLR2 ligand); poly(I:C), a synthetic analog of dsRNA (TLR3 ligand); LPA ultra-pure *Salmonella minnesota* LPS (TLR4 ligand); flagellin purified from *Salmonella typhimurium* (TLR5 ligand); and *CLO*97 imidazoquinoline compound (TLR7-TLR8 ligand).

### Clinical exome sequencing.

The GA4K is a large pediatric rare disease program at Children’s Mercy Research Institute in Kansas City that collects genomic data and health information for families with a suspected genetic disorder. The goal of GA4K is to expand diagnostic capabilities and catalog rare disease genomes and phenotypes within a health care system. Patients are referred from various specialties for inclusion in GA4K. After a patient is referred for the study, informed written consent is obtained from all participants prior to study inclusion. DNA extracted from the proband’s blood samples was used for whole-exome sequencing (WES) at the Center for Pediatric Genomic Medicine (CPGM) at Children’s Mercy Hospital. Exome libraries were prepared according to the manufacturer’s standard protocols for Illumina TruSeq library preparation and IDT XGen Exome Enrichment (Integrated DNA Technologies). Briefly, 250 ng high-quality genomic DNA (gDNA) was sheared by Covaris sonication to an average size of 450 base pairs. DNA fragments underwent end-repair, a-tailing, adapter ligation, and associated AMPure bead cleanups on a Hamilton NGS Star liquid handler. After adapter ligation, 10 cycles of standard PCR were performed with KAPA HiFi master mix (Roche) and primers specific to the ligated adapters, followed by a bead cleanup. The resulting libraries underwent quality assessment for the appropriate concentration and final library size. Six libraries were pooled together at 1,000 ng each for exome enrichment. Pooled libraries were blocked to prevent nonspecific binding and lyophilized with a vacuum concentrator. Pools were reconstituted with enrichment buffer (Integrated DNA Technologies [IDT]), standard exome baits, CNV baits, and custom mitochondrial baits. This exome enrichment reaction was subjected to a 4-hour hybridization, followed by incubation with streptavidin beads and serial washes with IDT’s enrichment wash buffers on a PerkinElmer SciClone liquid handler. An additional 10 cycles of standard PCR were performed with KAPA HiFi master mix and primers specific to the adapters, followed by a final bead cleanup. The resulting enriched pools underwent quality assessment for the appropriate concentration and final library size, as well as a TaqMan qPCR assay (Thermo Fisher Scientific) to ensure successful enrichment of target regions before standard Illumina Free-Adapter Blocking was performed. Cleaned, adapter-blocked pools were loaded onto an Illumina NovaSeq 6000 with a run configuration of 151 × 8 × 8 × 151. Resulting data were processed with Illumina’s bcl2fastq software. Sequence alignment and variant detection were performed with the Illumina DRAGEN Bio-IT Platform (versions 3.02–3.6.3) against the GRCh37 reference genome.

### Variant annotation.

Genetic variants with respect to hg19 were identified and imported into a proprietary annotation tool, Rapid Understanding of Nucleotide Variant Effect Software (RUNES). RUNES incorporated data from Ensembl’s Variant Effect Predictor software, produced comparisons with dbSNP, ClinVar, the Exome Aggregation Consortium (ExAC), gNOMAD (gnomad.broadinstitute.org), and known disease variants from the Human Gene Mutation Database (15, 36, 37). We focused our analysis on genes that are known to selectively increase susceptibility to HSE in adults or children, and other HSV-sensing genes (11, 38). RUNES reported allele frequencies (MAFs) derived from the CPGM’s Variant Warehouse Database. Potentially deleterious variants were identified using Variant Integration and Knowledge Interpretation in Genomes (15, 36, 37). As common variants (MAF >1%) are less likely to be functionally deleterious, we focused on rare variants (39). Biological context and prediction of deleterious functional effects were estimated using Polyphen-2 (http://genetics.bwh.harvard.edu/pph2/) and SIFT (40, 41).

### scRNA-Seq and PBMC sequencing.

PBMC samples from 32 individuals/pool were thawed and pooled proportionally on ice. Nuclei were isolated from an aliquot of 1 × 10^6^ pooled PBMCs following the 10x Genomics’ Nuclei Isolation for Single Cell Multiome ATAC + GEX Sequencing protocol using a lysis time of 3 minutes. After transposition, bel beads in emulsion were generated by loading 16,100 nuclei into each of 4 lanes of a Chromium Next GEM Chip J Single Cell (10x Genomics, 1000234), and libraries were prepared with the Chromium Next GEM Single Cell Multiome ATAC + Gene Expression Reagent Bundle (10x Genomics, 1000283) following the manufacturer’s protocol. Completed libraries were sequenced on an Illumina NovaSeq 6000. Gene expression (scGEX/scRNA) libraries were sequenced to a depth of 4.83 × 10^8^ reads per library with a run configuration of 28 × 10 × 10 × 90. Single-cell assay for transposase-accessible chromatin sequencing (scATAC) libraries were sequenced to a depth of 2.66 × 10^8^ reads per library with a run configuration of 52 × 8 × 24 × 52. Only single-nucleus (scRNA-Seq) data were used for analysis in this study.

### Analysis of scRNA-Seq data.

To process results from (RNA + ATAC) multiome sequencing experiments, we ran cellranger-arc (version 2) on all pools sequenced. From the cellranger-arc outputs, we determined the sample identity of each cell barcode with demuxlet using a genotype that had been imputed on the TopMed imputation server. Next, we used Seurat (version 4) and Signac in R to analyze the cellranger-arc/demuxlet results. Then, from the individual ATAC peak regions for all 88 captures, we created a common set of peak regions. We then applied QC filters to the cells (initial total of 761,929 cells) which: (a) kept only cell barcodes whose RNA and ATAC demuxlet identities were both classified as singlets (491,650 cells, 65% of the total), and (b) whose standard Seurat filters fell within a “normal” range (455,348 cells, 93% of the singlets). Once the individual captures were filtered, we split the data set into 2 sets (A and B) by randomly assigning half the captures of each pool to each set (227,350/227,998 cells in sets A/B). For each of the remaining steps, we did the analysis separately for data sets A and B. Using the common peak set that was previously created, we merged the Seurat objects from each capture into an aggregate. With the aggregate, we then created a new joint multimodal (RNA + ATAC) assay using the weighted nearest neighbor (WNN) method in Seurat. From the WNN graph, we then built a joint uniform manifold approximation and projection (UMAP) visualization. To determine cell-type clusters, we transferred cell-type labels to a previously studied multiomic PBMC data set. Then, we ran differential expression using Seurat to analyze the difference in RNA expression between sample cmh004121-01 and all other samples in the GA4K data set. This was done separately for each cell-type cluster. All data are deposited in dbGaP (https://www.ncbi.nlm.nih.gov/gap/) under the accession number phs002206.v4.p1. 

### Cell culture and transfection.

A commercial THP1 monocyte cell line (THP1-Dual cells) that had 2 inducible reporter constructs integrated, one for measuring NF-κB transcriptional activation and another for IRF3 transcriptional activity, was purchased from InvivoGen (catalog thpd-nfis). Cells were grown in RPMI 1640 media containing 10% FBS and antibiotics according to the manufacturer’s protocol in a humidified incubator containing 5% CO_2_ at 37°C. These cells stably express a NF-κB–inducible secreted embryonic alkaline phosphatase (SEAP) reporter gene that is induced upon the addition of bacterial TLR ligands, as well as an IRF3-inducible secreted luciferase upon stimulation with high-molecular-weight (HMW) poly(I:C) (InvivoGen), a TLR3 ligand. Both reporter proteins are readily measurable in the cell culture supernatant when using QUANTI-Blue, a SEAP detection reagent, and QUANTI-Luc, a luciferase detection reagent (InvivoGen). THP1-Dual was used to transfect the WT, mut, or mock (Ø) plasmids from *UNC93B1* and *IRF7* using Lipofectamine LTX Reagent with PLUS Reagent according to the manufacturer’s protocol (Thermo Fisher Scientific). Cells were used 48 hours after transfection. The HEK293T cells were used to transfect the WT, mut, or Ø plasmids from *IRF7* using Lipofectamine 3000 following the manufacturer’s protocol (Thermo Fisher Scientific).

### IRF3 activation and detection in THP1 cells.

We detected *IRF* activation with an IFN regulatory factor–inducible Lucia luciferase assay using the QUANTI-Luc detection system (InvivoGen) according to the manufacturer’s instructions. Briefly, cell culture supernatants (in triplicate) obtained from transfected THP1 cells 20 hours after poly(I:C) treatment were used. As quickly as possible, 20 μL supernatant was incubated with 50 μL QUANTI-Luc assay solution, the solution and supernatant were mixed by tapping the plate, and the plate was put into the plate reader. The luciferase was measured by the luminometer with a 0.1 second read time.

### Plasmid construction.

Human *IRF7* cDNA was amplified from pCMV3-IRF7 (SinoBioloical) with forward primer 5′-catGCTAGCcaccatggccttggctcctgagagggcag-3′ and reverse primer 5′-ctaCTCGAGctaggcgggctgctccagctccataagg-3′ and cloned into pIRES-EGFP-puro (Addgene) between NheI and XhoI. The G299C mutation (present in the most prevalent *IRF7* isoforms A and B, equivalent to G338C in isoform D) was introduced by 2-step PCR with 5′-CTTCCGCTGCGCACTGCCCAGCACGCGTCGCTTCG-3′ and its reversed sequence. Human *UNC93B1* cDNA was amplified from pUC-hUNC93B1 (SinoBioloical) with forward primer 5′-catGCTAGCcaccatggaggcggagccgccgccta-3′and reverse primer 5′-ctaGAGCTCtcactgctcctccggcccgtctcc-3′ and cloned into pIRES-EGFP-puro (Addgene) between NheI and SacI. The C1210T mutation was introduced by 2-step PCR with 5′-cacgcccggtgTccctggtggcc-3′ and its reversed sequence. FLAG-tagged WT or mut *IRF7* was amplified and subcloned into a lentiviral vector of pHIV-dTomato (Addgene) with 5′-CGTCTAGAACCATGGCCTTGGCTCCTGAGAG-3′ and 5′-CGGGATCCTTACTTATCGTCGTCATCCTTGTAATCGGCGGGCTGCTCCAGCTCCATAA-3′. For the construction of a lentiviral vector expressing both WT and mut *IRF7* and WT and mut *UNC93B1*, cDNA of *UNC93B1* WT or mut (without the stop codon) were inserted in front of FLAG-IRF7 interspaced with a P2A peptide (GGAAGCGGAGCTACTAACTTCAGCCTGCTGAAGCAGGCTGGAGACGTGGAGGAGAACCCTGGACCT). DNA sequencing was done to confirm our constructs (Eurofins Genomics). Expression of *IRF7* and *UNC93B1* was confirmed by Western blotting of cell lysates from HEK293T cells transfected with lentiviral vectors.

### Generation of HEK293T cell line for IRF7 nuclear binding studies.

HEK293T cells were purchased from Takara and grown in DMEM containing 10% FBS and antibiotics as per the manufacturer’s protocol in a humidified incubator containing 5% CO_2_ at 37°C. HEK293T cells were used to create a stable cell line for the WT and mut *IRF7* plasmids. Briefly, the lentiviral vector and packing vectors psPAX2 and pMD2.G were transfected into HEK293T cells using calcium phosphate transfection to produce lentiviral particles (42). After 2 days of transfection, supernatant containing lentiviral particles was harvested and used to transduce HEK293T cells to generate the stable cell lines 293T/Ø-dTomato, 293T/IRF7-FLAG-dTomato, and 293T/IRF7mut-FLAG-dTomato with comparable expression levels (Supplemental Figure 2).

### Generation of human neonatal fibroblast cell line for IRF7 and UNC93B1 WT and variant functional studies.

Newborn human foreskin fibroblasts were purchased from Coriell Institute for Medical Research (GM21808) and grown according to the manufacturer’s protocol in a humidified incubator containing 5% CO_2_ at 37°C. The fibroblasts stable cell lines were transduced with lentiviral particles carrying combinations of WT or mut *IRF7* and WT or mut *UNC93B1*. To increase the purity of the stable cell lines, the transduced fibroblast cells were sorted by flow cytometer according to their expression of red fluorescent dTomato protein. The cell lines generated were empty vector (Ø-dTomato), *IRF7* WT*:UNC91B1* WT, *IRF7:UNC93B1* mut, *IRF7* mut:*UNC93B1* WT, and *UNC93B1* mut:*IRF7* WT.

### IRF-Lucia luciferase assay.

The *IRF*-Lucia luciferase assay that is unique to the THP1-Dual monocytes was run following the manufacturer’s protocol (Thermo Fisher Scientific) and read on a luminometer plate reader. The assay is designed to monitor the activity of the IRF pathway by assessing the activity of the Lucia luciferase.

### IRF7 reporter luciferase assay.

The *IRF7* reporter luciferase assay examines the ability of WT and mut *IRF* alleles to induce *IRF7* transcriptional activity. We performed this assay using both transient transfection of *IRF7* alleles into HEK293T cells and into the 293T/Ø-dTomato, 293T/*IRF7*-FLAG-dTomato and 293T/*IRF7*mut-FLAG-dTomato cell lines we generated. PGL4-4X *IRF7*-minP-Luc was generated by inserting 4 copies of *IRF7* consensus–binding sites (ACGAAAGCGAAAGTACGAAAGCGAAAATACGAAAGCGAAAGTACGAAAGCGAAAAT) upstream of the minimal promoter-driven luciferase reporter of pGL4.29 (Promega) (43). pGL4-4X *IRF7*-minP-Luc, *Renilla* luciferase vector (pRL-TK), CFP-TLR3 (Addgene), and pIRES-Puro-EGFP-IRF7 (or mut) plasmids were cotransfected into HEK293T cells. The luciferase assay was applied using the Dual-Glo Luciferase Assay System (Promega) according to the manufacturer’s instructions after 2 days of transfection. In HEK293T stable cell lines expressing WT or mut *IRF7* alleles, we transfected pGL4-4X IRF7-minP-Luc, *Renilla* luciferase vector (pRL-TK), and CFP-TLR3. Two days after transfection, the luciferase reporter assay was performed in cell lysates.

### Propagation of HSV-1.

HSV-1 was purchased commercially from American Type Culture Collection (ATCC) (catalog VR260). The virus was propagated in Vero cells (CCL-81, ATCC) and titrated as previously described (44).

### HSV count in cells.

Human fibroblasts were seeded into a 6-well plate (2.0 × 10^5^/well) and infected with HSV-1 at a MOI of 10 for 1 hour, after which the media were exchanged for fresh culture media. Sixteen hours after infection, the fibroblasts were lysed with TRIzol (Thermo Fisher Scientific), and gDNA was isolated according to the manufacturer’s protocol (QIAGEN). The HSV-1 copy number and cell population were quantified by qPCR with the following HSV-1–specific primers and fibroblast primers: HSV-1 TK (forward), ATGGCTTCGTACCCCTGCCAT; HSV-1 TK (reverse), GGTATCGCGCGGCCGGGTA; fibroblast (forward), CCCGTGTTTAGCCTTGTTAAAG; fibroblast (reverse), CTAGGAATCCCGGACAGTTTG (45, 46).

### RNA and PCR assays.

RNA was harvested from THP1-Dual monocytes transfected with WT or mut *IRF*7 and *UNC93B1* variants using the TRIzol method, following the manufacturer’s protocol (Thermo Fisher Scientific). RNA was made into cDNA using iScript, following the manufacturer’s protocol (Bio-Rad). PCR for IFN-α and IFN-β was performed on a Bio-Rad CFX machine using primers manufactured by MilliporeSigma with SYBR (EVA) green from Biotium according to the manufacturer’s protocol.

### IP and Western blot assays.

THP1-Dual monocytes were transfected with WT *UNC93B1*, variant *UNC93B1*, or Ø plasmids. Cells were lysed with Pierce IP lysis buffer (Thermo Fisher Scientific) combined with protease and phosphatase inhibitors (MilliporeSigma). The clarified cell lysates were immunoprecipitated using protein G Surebeads (commercially available from Bio-Rad) following the manufacturer’s protocol, with a small aliquot taken out prior to IP for Western blot analysis. Western blots were run for protein analysis following standard protocols. IP was completed using mouse anti-TLR3 (Thermo Fisher Scientific, MA5-16184). The antibodies used for Western blot analysis were: rabbit anti-TLR3 (Abcam, ab13915), anti-UNC93B1 (Thermo Fisher Scientific, PA5-83437), mouse anti–IRF-7 (SCBT, sc-74471), and mouse anti–β-actin (MilliporeSigma, A1978). Densitometry was performed using ImageJ Software (NIH), and changes were normalized to β-actin or TLR3 (IP).

### IPA analysis.

Gene expression data from scRNA-Seq were obtained from differentially expressed genes and defined as a *P* value of less than 0.05 as assessed using an unpaired samples *t* test. The IPA system (version 73620684, Ingenuity Systems, QIAGEN) was used for subsequent bioinformatics analysis, which included canonical pathways, upstream analysis, diseases and functions, and regulator effects. For analyses, the –log(*P* value) >1.3 (*P* < 0.05) was set as the threshold, and a *z* score of ± 2 was defined as the threshold for significant activation and repression.

### Statistics.

Data are presented as the mean ± SD or the median with the IQR, and a *P* value of less than 0.05 was considered significant. For cell culture experiments, data from a minimum of 3 independent experiments with adequate technical replicates were used for quantification. RNA quantification and PCR were done with 2–3 technical replicates. Analysis was initially performed to determine whether the distribution of the data was Gaussian using the D’Agostino-Pearson omnibus normality test. If data were normally distributed, then ANOVA with a post hoc Tukey test was used for analysis. If data did not meet Gaussian assumptions, a 2-tailed Mann-Whitney *U* test was used for analysis. For most analyses, fold changes were calculated for the expression/changes in the Ø controls. Statistical analysis was done using Graphpad Prism 9.0 (GraphPad Software). For scRNA-Seq data, a Wilcoxon rank-sum test with Bonferroni’s correction was applied for multiple comparisons.

### Study approval.

Written informed consent was obtained from the parent of the proband prior to performing the studies. The proband’s immunological workup and exome sequencing were done as part of the clinical workup. In vitro functional studies were conducted under the approval of the Institutional Biosafety Committee of Children’s Mercy Hospital (protocol no. 00025). The patient was later referred and consented to the GA4K study for further genomic analysis. This study was approved by the Children’s Mercy IRB (study no. 11120514).

## Author contributions

MHT analyzed data, drafted the initial manuscript, and reviewed and revised the manuscript. WY planned and conducted experiments, acquired and analyzed data, wrote certain parts of the manuscript, and reviewed and revised the manuscript. The order of the co–first authors’ names was determined alphabetically. HM conducted experiments, acquired and analyzed data, drafted figures and figure legends, and reviewed and revised the manuscript. SX conducted experiments, acquired and analyzed data, and reviewed and revised the manuscript. CFS acquired and analyzed the single-cell data and reviewed and revised the manuscript. DL and MG were involved in performing the scRNA-Seq experiments and reviewed and revised the manuscript. TP conceived the scRNA analysis, supervised the scRNA-Seq experiments, and reviewed and revised the manuscript. NR analyzed the data and reviewed and revised the manuscript. VS conceptualized and designed the research studies, analyzed the data, drafted the initial manuscript, and reviewed and revised the manuscript.

## Supplementary Material

Supplemental data

## Figures and Tables

**Table 2:**
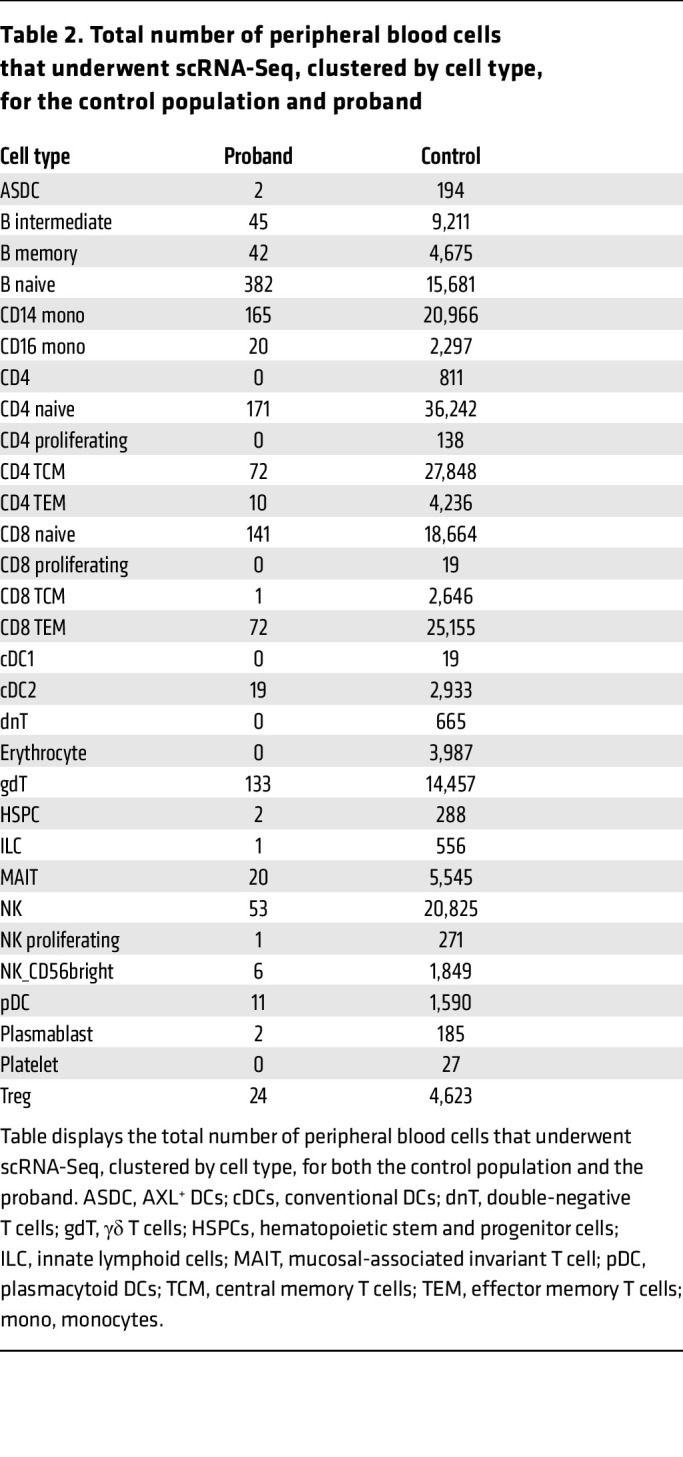
Total number of peripheral blood cells that underwent scRNA-Seq, clustered by cell type, for the control population and proband

**Table 1:**
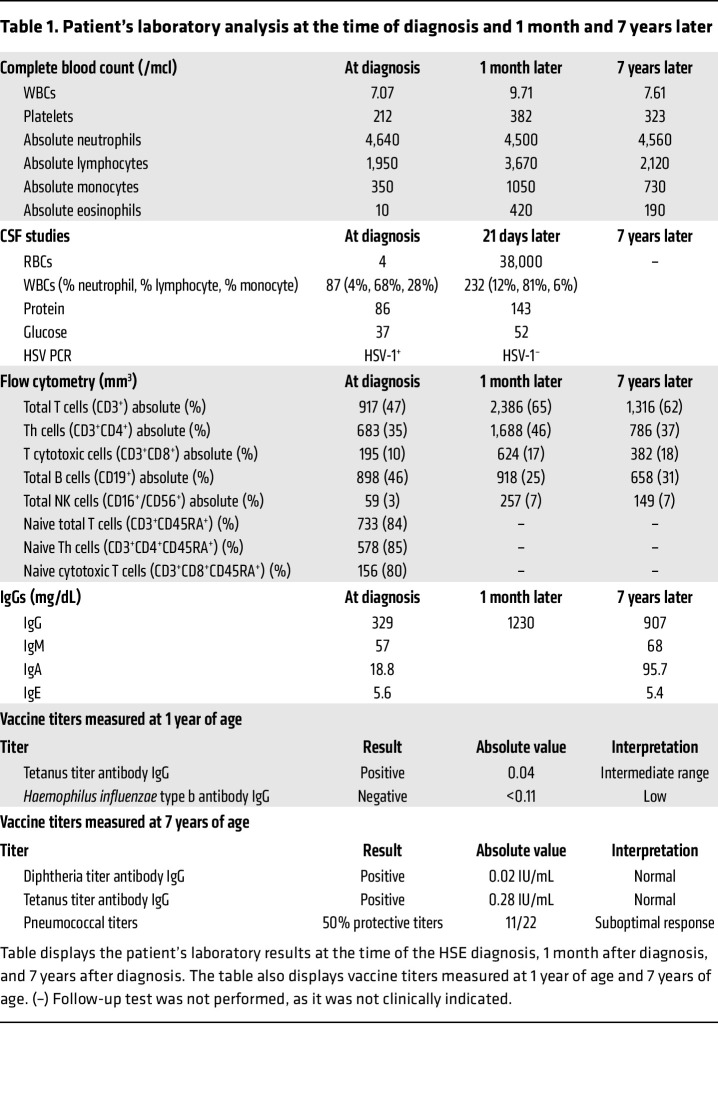
Patient’s laboratory analysis at the time of diagnosis and 1 month and 7 years later

## Version 1 04/25/2023

In-Press Preview

## Version 2 06/01/2023

Electronic publication
