# Supplementary material for: IRF7 and UNC93B1 variants in an infant with recurrent herpes simplex virus infection
Source: J Clin Invest. 2023 Jun 1;133(11):e154016. doi: 10.1172/JCI154016 (PMC10231989; doi:10.1172/JCI154016)
Supplement: Supplemental data [file jci-133-154016-s211.pdf]

Legends to Supplemental Documents

Figure 1 - Analysis of scRNA-seq transcriptome data in proband vs. controls: A) Depiction of genes induced in CD14, CD4 naïve, CD8 naïve, gDT, and CD4 naïve population in the proband compared to controls. **Note:** data shown is from only from approximately half of the cells of the control and half the cells of proband. The X-axis represents Log_2-fold gene expression, and the Y-axis represents Log_{10}-adjusted p value. **B-D)** IPA prediction of impaired TLR3 signaling in B cells (**B**), CD14 monocytes (**C**), and based gDT cells (**D**) based on altered expression of genes involved in TLR3 signaling. Shapes shaded in green represent downregulated genes with intensity of color proportional to the degree of downregulation. Shapes shaded in red/orange represent upregulated genes with intensity of color proportional to the degree of downregulation. Abbreviations: gDT cells, gamma delta T cells; IPA, ingenuity pathway analysis.

Figure 2 - Immunoblotting showing expression of IRF7 and *UNC93B1* alleles in HEK293T cells. Lentiviral vectors for both the reference and variant *IRF7* and *UNC93B1* alleles were constructed for fibroblast assays done in Figure 6. HEK293T cells were transfected with indicated lentiviral plasmids for 24 hours and cell lysates used to confirm expression of constructs by western blotting.

Supplementary Table 1: Table showing PBMC cytokine expression in response to various TLR ligands. Assay performed at ARUP laboratories (Salt Lake City, Utah, USA). Data show almost complete suppression of response to TLR3 [Poly(I:C)] stimulation with normal responses to other TLR ligands in comparison with simultaneously run controls. PBMC responses in a client control and reference laboratory control are shown. TLR ligands: PAM3CSK4 (TLR1, TLR2); Zymosan (TLR 2, TLR6); Poly (I:C) (TLR3); Flagellin (TLR5); CLO97 (TLR7, TLR8); LPS (TLR4). Abbreviations: CXCL10, C-X-C motif chemokine ligand 10; PAM3CSK4, Pam3CysSerLys4; Poly (I:C), polyinosinic:polycytidylic acid.

Supplementary Figure 1

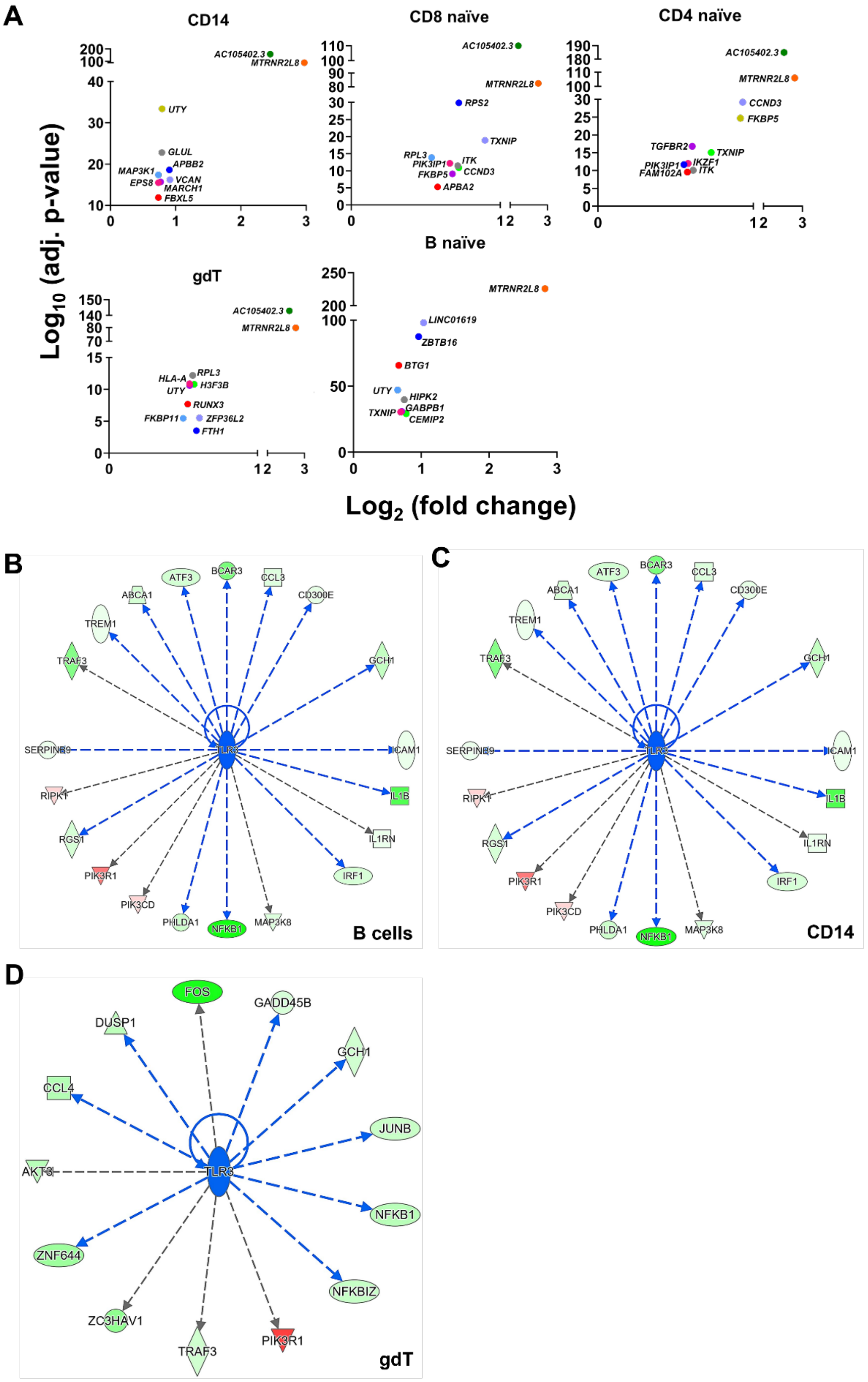

Supplementary Figure 2

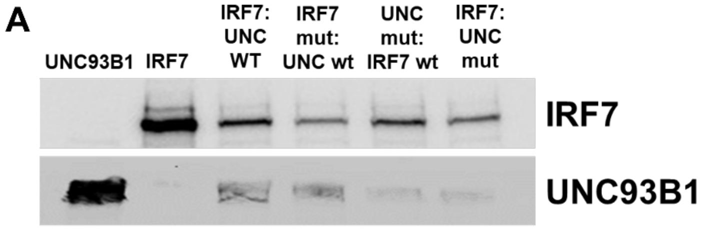

Supplementary Table 1

Toll-like Receptor (TLR) Functional Studies							
**TNF-a (pg/ml)**	**Patient**	**Client Control**	**Lab Control**	**IL-6**	**Patient**	**Client Control**	**Lab Control**
Media	0	4	4	Media	0	41	47
PAM3CSK4	492	931	1315	PAM3CSK4	10053	9461	10226
Zymosan	755	408	478	Zymosan	1641	586	713
Poly (I:C)	1	324	186	Poly (I:C)	11	3475	1512
Flagellin	1053	867	608	Flagellin	>10240	>10240	10207
CLO97	1984	2601	1827	CLO97	9769	>10240	>10240
LPS	2038	1764	1481	LPS	>10240	>10240	>10240
**IL-1b (pg/ml)**	**Patient**	**Client Control**	**Lab Control**	**CXCL10 (pg/ml)**	**Patient**	**Client Control**	**Lab Control**
Media	0	0	3	Media	4	30	56
PAM3CSK4	119	769	819	PAM3CSK4	N/A	N/A	N/A
Zymosan	571	776	755	Zymosan	N/A	N/A	N/A
Poly (I:C)	0	123	82	Poly (I:C)	9	5437	5187
Flagellin	222	988	419	Flagellin	N/A	N/A	N/A
CLO97	825	1915	1457	CLO97	N/A	N/A	N/A
LPS	438	3263	4147	LPS	N/A	N/A	N/A
